# Role of the Intestinal Microbiome, Intestinal Barrier and Psychobiotics in Depression

**DOI:** 10.3390/nu13030927

**Published:** 2021-03-12

**Authors:** Paulina Trzeciak, Mariola Herbet

**Affiliations:** Chair and Department of Toxicology, Faculty of Pharmacy, Medical University of Lublin, Jaczewskiego 8b Street, 20-090 Lublin, Poland; trzeciakp@onet.pl

**Keywords:** depression, mental diseases, gut microbiome, psychobiotics

## Abstract

The intestinal microbiota plays an important role in the pathophysiology of depression. As determined, the microbiota influences the shaping and modulation of the functioning of the gut–brain axis. The intestinal microbiota has a significant impact on processes related to neurotransmitter synthesis, the myelination of neurons in the prefrontal cortex, and is also involved in the development of the amygdala and hippocampus. Intestinal bacteria are also a source of vitamins, the deficiency of which is believed to be related to the response to antidepressant therapy and may lead to exacerbation of depressive symptoms. Additionally, it is known that, in periods of excessive activation of stress reactions, the immune system also plays an important role, negatively affecting the tightness of the intestinal barrier and intestinal microflora. In this review, we have summarized the role of the gut microbiota, its metabolites, and diet in susceptibility to depression. We also describe abnormalities in the functioning of the intestinal barrier caused by increased activity of the immune system in response to stressors. Moreover, the presented study discusses the role of psychobiotics in the prevention and treatment of depression through their influence on the intestinal barrier, immune processes, and functioning of the nervous system.

## 1. Introduction

Depression (major depressive disorder, MDD) is a serious medical illness that negatively affects thoughts, behavior, feelings, motivation, and sense of well-being [1]. Nowadays, depression is considered a civilization disease, due to its wide range and frequency of occurrence, especially in highly developed countries. Globally, about 300 million people, i.e., 4.4% of the world’s population, suffer from depression (Global Burden of Disease Study 2015). According to the Diagnostic and Statistical Manual of Mental Disorders (fifth edition; DSM-5), the diagnosis of major depression requires the presence of five or more symptoms within a two-week period [2]. One of these symptoms should be either a depressed mood or anhedonia (loss of interest or pleasure), whereas the others include appetite or weight changes, difficulty sleeping, diminished ability to think or concentrate, fatigue or loss of energy, feelings of worthlessness or excessive guilt and suicidality [3]. The multitude of observed cases of depression pose a challenge for researchers in terms of acquiring a deeper understanding of the etiology and mechanisms of depression. Since it is a disease related to the nervous system, most studies have focused on the search for biochemical and molecular bases of the disease, primarily in the brain structures involved with the onset of symptoms. Many in vivo and clinical studies have demonstrated the significant role of stress in the development of depression [4,5]. In addition to the above, a significant role has also been assigned to the immune system, which, in periods of excessively activated stress reactions, inter alia, negatively affects the tightness of the intestinal barrier and the intestinal microbiota. In turn, the intestinal microbiota has a significant impact on the functioning of the nervous system, including participation in the processes of neurotransmitter synthesis, myelination of neurons in the prefrontal cortex, and involvement in the development of the amygdala and hippocampus [6]. It is also known that there is a constant exchange of nerve and biochemical signals between the gut and the brain [7]. In view of the above, it seems very important to present and link these aspects in the pathomechanism of depression.

The available literature lacks data on simultaneous abnormalities in the functioning of the intestinal barrier, caused by increased activity of the immune system in response to stressors. The literature also lacks data on the influence of the intestinal microbiota on the nervous system as a consideration of the multidirectional factors influencing the manifestation of the depression. A consequence of examining the mechanisms related to the pathophysiology of depression, is the search for new drugs and therapeutic strategies for this disease, since the current treatment methods—although having been slowly supplemented with new drugs in recent years—are still unsatisfactory [8]. Most of the commonly used medications only alleviate the symptoms of the disease and are often ineffective and burdened with numerous side effects. In recent years, research has been conducted on probiotic bacteria (psychobiotics), which—when consumed in appropriate amounts—have a positive effect on mental health, but so far have not been included in the treatment of mental diseases [9]. In the presented work, the role of psychiobiotics in the prevention and treatment of depression will be discussed in detail through their influence on the intestinal barrier, immune processes, and functions of the nervous system.

## 2. Neurophysiological and Neurochemical Aspects of Depression

Although the mechanisms of depression are not yet fully understood and explained, several theories detailing the appearance of this disease have been published and thoroughly described [8]. Brain serotonergic, noradrenergic, and dopaminergic transmission significantly affect the functioning of the central nervous system (CNS). Transmission of monoamines is responsible for mood, cognitive functions, sleep regulation, and stimulation of the reward center. The monoamine theory is supported by monoamine reuptake inhibitors that have been shown to be effective in treating depression. In depression, the concentration of 5-hydroxyindole acetic acid (5-HIAA), the main metabolite of serotonin (5-HT) in the cerebrospinal fluid, decreases [10,11,12,13,14]. This pathophysiological change may result from two mechanisms. The first, involves a reduction in the availability of 5-HT, with a consequential compensatory upregulation or oversensitivity effect on receptors. The second, implies a primary defect in receptor activity and/or signal transduction. Selective serotonin reuptake inhibitors (SSRI) currently represent the group of medications most commonly prescribed to treat depression. They are often prescribed as first-line pharmacotherapy for the treatment of depression because of their efficacy, safety, and tolerability. The action of the serotonin system may have an effect on sleep, sexual activity, appetite, and target-directed actions [15]. Studies using the selective 5-HT1A receptor agonist—ipsapirone—also testify the decreased serotonin transmission in patients with depression. This substance, when administered to depressed individuals, causes a weaker response than in healthy people. Additionally, excluding the precursor for the production of serotonin—L-tryptophan—from an individual’s diet results in a lack of response to treatment with SSRI [16]. Conducted studies also show an increased sensitivity of 5-HT2 receptors. Serotonin 5-HT1A receptors in patients with depression are characterized by a decrease in sensitivity which has been confirmed in research [17].

Increased concentrations of noradrenaline (1-(3,4-dihydroksyfenylo)-2-aminoetanol, NA), adrenaline (4-[(1S)-1-hydroxy-2-(methylamino)ethyl] benzene-1,2-diol, A) and their metabolites have been recorded in patients with severe depression. The reason for this may be the stimulation of the hypothalamic–pituitary–adrenal axis (HPA) [18]. Studies also indicate changes in the density and sensitivity of β and α2 adrenergic receptors [15]. Dopamine (3-Hydroxytyramine, DA) also plays a role in the pathogenesis of depression through the mesolimbic and mesocortical systems, which are responsible for negative cognitive symptoms, reduced pleasure, and motor inhibition [11]. As in the case of serotonin and noradrenaline, the concentration of the main dopamine metabolite, homovanillic acid (HVA), is reduced in patients with depression [19]. The role of dopamine in the pathomechanism of depression has also been confirmed by studies of learned helplessness in animals, which have shown reductions in dopaminergic transmission in the mesolimbic system [20]. Disturbances in dopaminergic transmission are corroborated by the effectiveness of dopamine agonists in the treatment of symptoms of drug-resistant depression. Studies on patients with depression have also shown increased levels of glutamate (2-aminopentanedioic acid, GLU) in the cerebrospinal fluid [21]. Overactivity of the glutamatergic system—the cause of which may be chronic stress—causes degenerative changes in nerve cells. It is believed that this action of glutamate may be involved in the pathomechanism of depression. Participation of this system in the development of depressive disorders seems to be confirmed by the results of tests using NMDA (N-methyl-D-aspartate) receptor antagonists. It turns out that their use has a quick antidepressant effect [22]. Some studies have also been conducted to explain the role of γ-aminobutyric acid (GABA) in the pathogenesis of depression. Lowered GABA levels in the nervous system have been shown in depressed patients in comparison to healthy controls [23]. Another neurotransmitter involved in the pathomechanism of depression is acetylcholine (choline acetate, ACh) [24], the constitutive compounds of which—with cholinomimetic activity—cause symptoms similar to those present in depression, or reduce the symptoms of mania in patients with bipolar disorder. On the other hand, agents with anticholinergic activity are characterized by antidepressant activity and induce the symptoms of mania [25]. Additionally, it is believed that the increased activity of cholinergic neurons may increase the activity of the HPA. In the pathomechanism of depression, attention has also been paid to the function of secondary transmitters and transcription factors including—among others—cAMP (cyclic adenosine monophosphate), the level of which increases after the use of antidepressants.

Increasingly, dysfunction of endocrine mechanisms is considered to be a possible cause predisposing to the development of depression. Dysregulation of the HPA increases the risk of affective and anxiety disorders [4]. Stress activates the HPA system, and the first stage of the body’s incipient response to a stressor is the increased release of corticoliberin (CRH) in the hypothalamus. The hypothalamus cooperates with the pituitary gland and the chemical signals that reach the pituitary gland from the hypothalamus are decisive. The signal that stimulates the pituitary gland to release pituitary corticotropin in a stress response is the aforementioned CRH. The functioning of the hypothalamic–pituitary–effector organ axis, as a rule, dictates the existence of subordinate organs. In a stress reaction, these are the adrenal glands which, following the order of the decision-making organs, secrete significant amounts of cortisol. Cortisol causes a follow-up effect in the form of mobilization of the body to adopt the so-called fight or flight mechanism [26]. In depression, excessive activation of the HPA axis occurs due to the increased impact of CRH and damage to the feedback mechanisms [4]. Such a significant impact of stress not only affects the parameters and functions of the cardiovascular system, but also the immune and digestive systems. Currently, normalization of the HPA axis is a determinant for the search for new generation antidepressants [27].

Oxidative stress and inflammatory processes are also involved in the pathomechanism of depression, associated with chronic stress [28]. The pathological influence of oxidative stress is the formation of unpaired electron compounds. Under physiological conditions, these compounds are neutralized [29]. However, if not inactivated, they can inhibit enzyme activity, cause lipid peroxidation, or damage DNA (deoxyribonucleic acid) and consequently lead to apoptosis or necrosis of cells [30]. It is believed that one of the effects of oxidative stress is decreases in the numbers of nerve and glial cells in the CNS [28]. Studies have shown that in patients with depression, the activity of catalase— one of the most important antioxidant enzymes—is reduced. Increased concentrations of oxidative stress markers, such as MDA (malonyldialdehyde), has also been recorded [29]. In patients suffering from depression, an increased concentration of omega-3 acids was also detected, which may suggest damage to biological membranes. The oxidative–reduction balance, in turn, plays a role in the modulation of inflammation and the immune response. Excessive production of intracellular reactive oxygen species contributes to triggering the inflammatory response through the secretion of pro-inflammatory mediators, such as leukotrienes, prostaglandins and cytokines [31]. In people suffering from depression, increased levels of interleukins: IL-6 and IL-1, as well as TNFα (tumor necrosis factor α) and interferon γ have been found. Research conducted in rodents indicates that administration of IL-1 causes depressive disorders, including withdrawal from social behavior and decreased sexual activity [32]. The opposite effect was achieved by deleting the genes encoding Il-6 or TNFα and blocking the receptors for Il-1 [33].

An important consideration is that depression can have a relationship with neuroplasticity. The brain derived neurotfrophic factor (BDNF) is responsible for the formation of new connections between neurons—a brain factor of trophic origin [34]. Many studies have found that this neurotrophin influences the development of serotonergic, dopaminergic, noradrenergic and cholinergic connections [35,36,37,38]. BDNF has the ability to cross the blood–brain barrier (BBB) [39], therefore, it is suggested that its concentration in blood plasma may represent its concentration in the brain and that both concentrations could function as the same diagnostic marker. The above statement was corrected by studies which showed a positive correlation between the concentrations of BDNF in the serum and in the cortex of rats [40]. Research on the peripheral concentration of BDNF in patients suffering from depression has been conducted since the beginning of this century. It was determined that the concentration of BDNF in the blood plasma was much lower in the group of untreated patients compared to the group of treated people and the control group [41].

## 3. The Role of the Intestine Microbiome in Depression

One of the newest theories of the pathophysiology of depression focuses on research into the gut microbiome [42]. It has been observed that the gut and brain work in a bi-directional manner and can affect each other’s functions and significantly impact stress and depression [43]. A healthy gut microflora is known to transmit signals to the brain via pathways involved in neurotransmission, neurogenesis, microglia activation, and behavioral control under both normal and stressful conditions. Studies into the effects of the gut microbiota on behavior and neurobiology, called the microbiome–gut–brain axis (MGBA), began with the observation of patients suffering from inflammatory bowel disease and irritable bowel syndrome (IBS) [44,45,46]. It has been noticed that the composition of the intestinal microflora in patients with depression differs from that in healthy people, as confirmed in animal models of depression [47,48]. The influence of intestinal microflora on depressive behavior was also demonstrated in studies carried out on rats and mice born and raised in a microflora-free environment and in animals with specific pathogen-free (SPF) gut microbiota [49,50,51]. Colonization of pathogen-free animals with SPF intestinal microbiota has been shown to ameliorate their behavior [52,53]. Some bacteria have been observed to produce neuromodulatory substances such as those found in the nervous system of animals: acetylcholine, dopamine, serotonin, GABA, norepinephrine [54,55].

The adult human gut microbiome includes about 10^13^–10^14^ microorganisms, including: bacteria, viruses, fungi, archaea, and protozoa [56]. The composition of the microbiota is unique for each individual and is the result of various factors related to changes in the intestinal environment, lifestyle, and dietary habits [57]. The functions of the gut microbiota can be defined in three categories, i.e., metabolic, trophic, and protective functions. The metabolic function is carried out by decomposition of undigested food residues and production of B vitamins and vitamin K [58]. The trophic functions include controlling the tightness of the intestinal epithelium by participating in processes related to the maturation and exchange of enterocytes, while the interaction of the microbiota in terms of activity is another example of gastrointestinal (GI) motor skill functioning [59,60,61].

Intestinal bacteria are also a source of vitamins, including vitamin K-2 and B vitamins (niacin, biotin, folic acid, and pyroxidine) [62,63,64]. Studies have shown low levels of folate in the blood serum of patients with depression. This deficiency is believed to be related to the response to antidepressant therapy and may lead to exacerbation of depressive symptoms [65]. In turn, pyroxidine is an essential cofactor of enzymes that are altered in people with depression. Such enzymes are involved in the kynurenine pathway and depressed individuals have increased susceptibility to pyroxidine deficiency, which is demonstrated by disease-free animals [66].

Despite the significant role attributed to microorganisms that make up the microbiota, it is believed that this environment also affects the immune and nervous system. Each permutation in the composition of the gut microbiome results in the production of lipopolysaccharides (LPS) by the microorganisms, which in turn activates inflammatory responses. The produced cytokines send signals to the vagus nerve, thus, connecting to the HPA axis. Behavioral effects are a consequence of these processes. It has also been shown that GI inflammation can lead to inflammation of the nervous system, which in turn drives the action of microglia, triggers the kynurenine pathway and, consequently, may contribute to the development of depression [67]. Most importantly, this influences the production of pro-inflammatory cytokines and the functioning of the nervous system through participation in the synthesis of neurotransmitters. The synthesis of GABA, serotonin, glutamate and BDNF is important in affecting the nervous system [6]. Over the course of several years, it has been determined that the microbiota influences the shaping and modulation of the functioning of the gut–brain axis. Studies carried out with germ-free (GF) mice and rats (animals kept in sterile conditions, without intestinal microbiota) have shown that the intestinal microbiome has a significant influence on the formation of neural networks of the enteric nervous system (ENS), as well as the neuronal connections between the ENS and the central nervous system (CNS) [68]. In the early stages of life, a lack of specific microbes in the gut results in an overactive stress response later in life. [69]. Importantly, the intestinal microbiota is involved in the processes of myelination of neurons in the prefrontal cortex and is involved in the development of the amygdala and hippocampus [35].

Research on the intestinal microbiota has brought a new perspective on the pathomechanism of many diseases. In the context of depression, it has become a topic of interest for determining the composition of the microbiota in those suffering from this disorder. Indeed, many studies have indicated that people with depression are disturbed by both the composition and the number of strains that make up the gut microbiome [70].

Liu et al. evaluated the gut microflora of 90 young American adults by comparing the intestinal microflora of 43 participants with (MDD) and 47 healthy individuals in the control group. The study found that people with MDD had significantly different gut microbiota compared to the control group. People suffering from MDD had lower levels of *Firmicutes* and higher levels of *Bacteroidetes*, with similar trends in class (*Clostridia* and *Bacteroidia*) and row (*Clostridiales* and *Bacteroidales*). At the genus level, the MDD group showed lower levels of *Faecalibacterium* and other related members of the *Ruminococcaceae* family, which were also lower compared to healthy controls. In addition, participants with MDD enriched the *Gammaproteobacteria* class. The study authors conclude that the difference in the abundance of these bacterial strains resulted in a reduced ability to produce short-chain fatty acids (SCFA) in people with MDD [71].

In a separate study, Huang et al., using rRNA 16S sequencing and bioinformatics analysis, assessed the composition of the gut microbiota. The study material consisted of fecal samples taken from 54 people (27 patients with MDD). The results showed that patients with depression have a serious disorder of the composition of the intestinal microbiota. The authors observed a significant decrease in the amount of *Firmicutes* [72]. Analysis of the results of the two above studies led to the conclusion that reducing the amount of *Firmicutes* results in a decrease in SCFA. *Firmicutes* bacteria contribute to the fermentation of carbohydrates into SCFA [73]. It is claimed that SCFA deficiency may weaken the function of the intestinal barrier [74]. It is noteworthy that the leakage of the intestinal barrier contributes to pathogens and their metabolites crossing the barrier This process induces an immune response, which may be linked to the occurrence and development of depression [75]. To confirm this relationship, a separate study was carried out. This study eventually showed a significant correlation between stress-induced behavioral changes in mice and *Firmicutes* disorder in the gut microflora [76]. With the decline of *Firmicutes*, the protective factors of the intestinal barrier weaken and the body is additionally exposed to the risk of inflammation [72].

The comprehensive meta-analysis in patients with MDD has indicated that several taxa at the family and type level were reduced, particularly within the *Prevotellaceae*, *Corprococcus* and *Faecalibacterium* families compared to the control group. The study also confirmed the beneficial aspect of using probiotics, which improved the symptoms of depression [77]. A separate meta-assessment of adults over 18 years of age suffering from MDD and healthy adults, reviewed disorders in the composition of the microbiota. Differences in α and β microbiota occurred in people with depression compared to healthy control subjects at the level of the *Bacteroidetes, Firmicutes* and *Proteobacteria*. The high abundance of *Fusobacteria* and *Actinobacteria* was observed in people suffering from depression. In patients with depression, the high abundance of *Actinomycineae*, *Bifidobacteriaceae*, *Clostridiales incertae sedis*, *Clostridiaceae*, *Eubacteriaceae*, *Fusobacteriaceae*, *Lactobacillaceae XI*, *Nocardiaceae*, *Porphyromonadaceae*, *Streptomycetaceae*, *Thermoanaerobacteriaceae* and low abundance of *Bacteroidaceae*, *Chitinophagaceae*, *Marniabilaceae*, *Oscillospiraceae*, *Streptococcaceae*, *Sutterellaceae* and *Veillonellaceae* at the family level was observed. In turn, at the genus level, a high abundance of *Actinomyces*, *Anaerofilum*, *Anaerostipes*, *Asaccharobacter*, *Atopobium*, *Blautia*, *Clostridium IV*, *Clostridium XIX*, *Desulfovibrio*, *Eggerthella*, *Erysipelotrichaceae incertae sedis*, *Eubacterium*, *Gelria*, *Holdemania*, *Klebsiella*, *Olsenella*, *Oscillibacter*, *Parabacteroides*, *Paraprevotella*, *Parasutterella*, *Parvimonas*, *Streptococcus*, *Turicibacter*, *Veillonella*, and low abundance of *Clostridium XlV*, *a Coprococcus*, *Dialister*, *Escherichia/Shigella*, *Lactobacillus*, *Howardella*, *Pyramidobacter* and *Sutterella* was found in depressed patients [78].

## 4. The Role of Metabolites of the Intestinal Microbiome in Depression

Nutrients derived from food can be metabolized into small-molecule compounds such as SCFA, indoles and its analogues, or acortic acids. The above compounds do not only elicit local actions, but also affect distant tissues and organs [79]. Metabolites of the intestinal microbiota are a source of energy for colonocytes and, by acting as nuclear receptors, modulate a number of immune processes and affect the course of inflammatory reactions and the synthesis of neurotransmitters. There is a great deal of evidence that the above products have a potential association with the occurrence of depression [80,81,82]. Products of metabolism of the intestinal microbiota also include SCFA. With the participation of bacteria in the GI tract, acetate, butyrate and propionate are formed, which—according to research—yield promising benefits. These compounds are linked to the occurrence of pain, depression or neurodegenerative diseases. The above action is realized through the participation of SCFA in anti-inflammatory processes. SCFA interact with NLRP3 (NOD-like receptor family, pyrin domain containing 3) inflamasome cells in the intestinal epithelial cells. This relationship increases the production of IL-8 and improves the tightness of the intestinal barrier [83,84]. The cells of the immune system are also targeted by SCFA. Butylan and propionate have been shown to inhibit the formation and differentiation of dendritic cells, which are responsible for immune dysfunctions [85]. The study of transplantation *Bacterioides thetaiotaomicron* (manufacturer of acetate) to GF mice can promote the production of mucin and affect the integrity of the intestinal barrier. SCFA may reduce the production of pro-inflammatory cytokines from neutrophils and lipopolysaccharide activated macrophages (LPS) by inhibiting HDAC (histone deacetylases) [86]. Such a broad effect of SCFA affects their involvement in eliminating pain reactions and depressive states. In addition, butylan has the potential to maintain the integrity of the BBB. It should be emphasized that colonization with a bacterium producing buttermilk (*Clostridium tyrobutyricum*) and oral administration of sodium buttermilk (1000 mg/kg for 3 days) can fix the BBB leak. These changes are related to increasing the expression of proteins with close connections [87]. Based on new research determining the role of buttermilk, it is claimed that this compound affects the behavior, memory and levels of neurotrophic factors in a rat model of chronic mild stress. This study shows antidepressant effects [88]. In addition to the effect on the regulation of neurotrophic factors, butyric acid usually inhibits the deacetylation of histones and prevents activation in the hippocampus. Butyrate, which is a by-product of the metabolism of intestinal bacteria modulates the synthesis of dopamine and norepinephrine. Butyrate affects the change in the expression of the gene that encodes tyrosine hydroxylesis [89].

One of the fine-particle products of microbiota metabolism is secondary bile acids. Bile acids are synthesized from cholesterol in the liver and then metabolized into other bile acids by colon bacteria through numerous enzymatic pathways [90]. Secondary, cholic acids formed by the use of intestinal bacteria include lithocholic acid, deoxycholic acid and ursodeoxycholic acid. These compounds are formed by decoumination and 7α-dehydroxylation [91,92]. Over the years, scientific studies have shown that bile acids have extensive physiological effects, elicited by activating specific receptors in the nucleus and cell membrane [91]. These receptors can mediate various pathophysiological processes, including glucose homeostasis, and inflamed and sensory transduction. Bile acids can affect nuclear receptors (farnezoid X receptor (FXR), preganate X receptor and vitamin D receptor) and surface receptors (for example, bile acid receptor coupled with G protein—GPBAR1 (G-Protein Coupled Bile Acid Receptor 1)) or TGR5, sphingosine phosphate receptor 2 and musculature receptors 2 and 3). In the case of pathogenesis of depression, activation of the FXR ligand is particularly important. A recent study showed that over-expression of hippocampal FXR causes depression-like symptoms and reduces BDNF expression in the hippocampus in naïve rats [92]. Similarly, treatment with tauroursodeoxycholic acid could prevent the depressive behavior caused by LPS. This possibly occurs through weakening neural system inflammation and oxydonitrozative stress [91]. In this regard, it has been shown that the inhibition of nuclear glycelic factor-κB (NF-κB) and the activation of TGR5 in microglial mediates the effect of tauroursodeoxycholic acid on the production of pro-inflammatory cytokines [93].

The metabolism of the intestinal microbiota is associated with the existence of many pathways, metabolic processes and assumes the activity of many enzymes. A wide range of biochemical metabolites, formed by transformation, affects the functioning of the human body. In addition, the wide role of the microbiota also assumes its involvement in the processes of modification of amino acids. Many scientific publications indicate that tryptophan (TRP) is a key amino acid associated with the metabolism of the gut microbiota. Bacteria of the *Firmicutes*, *Clostridium sporogenes* and *Ruminococcus gnavus* families convert TRP into a biogenic amines after birth. These amines are structurally similar to serotonin. It is worth noting that this reaction occurs using the tryptophan decarboxylase enzyme [94]. *Ruminococcus gravus* is a common bacterium and is common in adults [95] and infants [96]. Tryptamine is a product of metabolism that maintains normal intestinal homeostasis [97].

Nevertheless, the most important metabolite of the intestinal microbiota is indole, which is wiped out by many Gram-positive and negative bacteria [98]. Indole and its derivatives are produced by the bacterial enzyme tryptophanase [99,100,101]. The above compound is a signaling molecule that stimulates enteroendocrine L cells to “separate” glucagon-like peptide 1 (GLP-1). GLP-1 in turn stimulates the aferent activity of the vagus nerve in the colon [102,103]. Indole also regulates the permeability of the intestinal barrier [104]. In the context of behavioral disorders, excessive amounts of indole cause a negative effect, increasing anxiety and depressive behaviors in rats. In addition, indole is associated with the sensitivity of mice exposed to chronic stress and interferes with the biosynthesis of catecholoamins in the adrenal core [105]. Jaglin et al. determined the effect of indole on physiology and behavior in rats [106]. Following acute administration, there was a significant reduction in the mobility and accumulation of indole metabolites in the brain. This study suggests a possible effect of indole on central receptors. Chronic exposure to indole, achieved by the colonization of GF rats by *E. coli*, increased anxiety and helplessness behavior (i.e., depressive behavior). On the other hand, separate studies have shown that indole and its derivatives (e.g., Indoxyl-3-sulphate (I3S), indole-3-propionic acid (IPA) and indole-3-aldehyde (IAld)) are capable of activating the aryl hydrocarbon receptor (AhR). This action has a subsequent inhibitory effect on the nervous system [107]. Rothhammer et al. demonstrated that neural system inflammation was reduced by activating AhR on astrocytes in mice. These mice were either supplemented with indole or related compounds [108]. These activities described above make it difficult to understand the physiological and pathological role of indole. The reason for this, is the existence of a large number of indole derivatives with diverse and dynamic effects [109].

Studies on animal models have shown that the gut microbiota affects the levels of amino acids in the blood. This also has an impact on the occurrence of depression. Analysis of the fecal metabolome in rats exhibiting depressive behavior revealed changes in AA levels of L-treonine, isoleucin, alanine, serine, tyrosine and oxidized proline. Changes in amino acid levels in the plasma—correlated with both the phylogenetic composition of bacteria and changes in amino acid levels—were observed in fecal metabolomy [110]. In view of the above, it is considered that metabolites of arginine catabolism have an effect on depression. Many studies have highlighted the antidepressant and anxiolytic effects of putrescin and agmatine. These compounds naturally occur as a result of arginine decarboxylation [111].

Additionally, a product of microbiota metabolism is lactate, which is formed by the fermentation of dietary fiber by lactic acid bacteria (e.g., *Lactobacillus lactis, Lactobacillus gasseri* and *Lactobacillus reuteri*), *Bifidobacteria* and *Proteobacteria*. [112] Lactate is used as an energy substrate by neurons in the brain because it crosses the BBB [113]. In addition, its ability to convert into glutamine contributes to the formation of synaptic plasticity. This plasticity is crucial in the formation of memory pathways [114,115,116]. In the context of depression, both animal model studies and clinical trials have determined that there is an apparent relationship between lactate disorders and the onset of symptoms of depression. Thus, elevated levels of lactate in urine were observed in patients suffering from severe MDD compared to the control group [117]. Carrard and others (2018) have also demonstrated the antidepressant effects of acute and chronic intraitoneal L-lactate injections in a mouse model of depression with corticosterone. These behavioral effects followed an increase in L-lactate concentrations in the hippocampus and were dependent on changes in the expression of several genes associated with depression pathophysiology [118].

## 5. The Intestinal Barrier as a Link between the Gut Microbiome and the Brain

Anatomically, the digestive system is connected to the CNS by nerve cells and fibers [7]. This system works with the coating, immune, endocrine system, microbiota and creates the so-called intestinal barrier. The intestinal barrier is a structure that includes the aforementioned microbiota and mucus-producing intestinal epithelial cells connected by tight junctions [119]. One of the building blocks of the intestinal barrier is the lamina propria, which contains cells of the immune, lymphatic, nervous and blood systems. Many scientific sources confirm that the intestinal barrier is an element that is closely related with the intestinal microbiota [120,121,122]. In a pathological state the, dysfunctions of the intestinal barrier and the modified composition of the microbiota are associated with the occurrence of chronic inflammations of the GI tract. Still, little is known about the effect of the intestinal microbiota on the intestinal barrier [123]. While the literature mentions that the microbiota affects mucosal and systemic immunity, while mediating the proper architecture of the intestinal barrier, the etiology of the above relationships cannot be fully explained [124,125]. For this purpose, one study compared the intestinal environment of GF mice, conventional mice, as well as mice colonized with human fecal microflora [126]. Observations were made for 21 days after colonization. The structure of the colon barrier was studied using immunohistochemical techniques, in addition to molecular and electron microscopy. Permeability was assessed in the colon tissue using chambers and by detecting serum LPS and MDP (muramyl dipeptide) using TLR4- and NOD2-NFκB (nucleotide-binding oligomerization domain 2—nuclear factor-kappa B) reporter tests. The microbiota profile was studied by sequencing the Illumina 16S rRNA gene. The experiment also reported a low dose of dextran sodium sulphate (DSS) to assess barrier changes caused by the microbiota for resistance to colon damage. The permeability of the paracellular probes and mucus layer structure resembled that of conventional mice on the seventh day after colonization, which coincided with reduced claudine-1 expression and transient production of IL-18 by intestinal epithelial cells. Adaptive changes after colonization were associated with reduced systemic exposure to bacterial antigens and reduced susceptibility to intestinal damage. The authors of the study found that commensal colonization promotes structural and functional adaptations of the physiological barrier that contribute to the preservation of intestinal homeostasis. Explaining the above mechanisms, it was found that—compared to conventional SPF mice—GF mice had a smaller paracellular uplift of an inert probe in the proximal colon. This fact suggests that the microbiota is necessary to establish certain semi-permeable paracellular characteristics of the colon [127]. In another study, to show the dynamics of changes in the intestinal barrier, adult GF mice were colonized with the fecal microbiota of a healthy donor and then subjected to intensive screening for the absence of common pathogens [128]. The colonization was carried out outside the postpartum period to avoid age-related developmental and dietary changes [128]. Paracellular colon permeability reached physiological status within a week of colonization. This was not related to mucosal damage and was independent of the colonization mode, suggesting that this was not due to an overt inflammatory response to microbial inoculum or gastric gavage surgery. Increased systemic exposure to bacterial immunostimulants occurred before paracellular permeability had achieved a physiological state. According to the researchers, this refers to the increased bacterial exposure. When mucus integrity is low, this state allows passage of entities through the “leakage” path of a leaking joint [129]. Therefore, the results of the above studies confirm that the microbiota plays an overarching role in the process of strengthening the mucus, and thus, ensuring the tightness of the intestinal barrier [130,131,132,133]. The gut microbiota continuously affects the function and integrity of the barrier. The disturbed continuity of the barrier can predispose to the development of dysfunction and chronic intestinal disorders in adulthood [134,135]. The intestinal barrier depends on the coordinated contribution of a complex network of cellular, immunological, biochemical, or microbe factors. Moreover, this monolayer, closely connected by the help of close cylindrical epithelial cell connectors, confers a selective barrier function [136]. It is well known that commensal gut bacteria can have a profound impact on epithelial permeability and integrity, in particular, on the repair and maintenance of the abovementioned contact links [137,138,139,140,141]. Mao et al. demonstrated that human *L. plantarum* and the rat-derived *L. reuteri* strain may reduce permeability dysfunction in the methotrexate-induced colitis model in rats [142]. Interestingly, this study showed that rats given *L. plantarum* exhibited fewer *Enterobacteriaceae* and Gram-negative anaerobic infections in the boules [143]. According to many studies, various growth factors, including transformative growth factor (TGF)-α and TGF-β, epidermal growth factor (EGF), insulin-like growth factor (IGF), hepatocyte growth factor (HGF), keratinocyte growth factor (KGF), fibroblast growth factor (FGF), cytokine, interleukin (IL)-1β and IL-2 and trefoil peptides increase the proliferation of colon epithelial cells [144,145]. It is noteworthy that the GF mice show a decreased level of TGF-p expression compared to the conventional mice. Several species of *Clostridium*, *Bifidobacteria* and *Lactobacilli* also exhibit a tendency to modulate cellular functions mediated by TGF-β, KGF and EGF in colon epithelial cells [146]. In the physiological state, the intestinal microbiota affects the processes of cell proliferation, ensuring the accuracy of the cells of the colon epithelium. In addition, the microbes affect the formation of expression of certain growth factors, and through the production of metabolites, contribute to the maintenance of homeostasis of the intestinal environment.

A well-functioning barrier enables proper cooperation between the HPA and the intestines. All processes which result in the stimulation of the nervous system are mediated by cytokines [147]. The interaction of cortisol with mast cells helps to release cytokines from their granules, which detoxify the protein structures of tight junctions. The presented activity contributes to the unsealing of the junctions between enterocytes and ultimately contributes to an increase in the permeability of the intestinal barrier. The de-encapsulation of the dam results in undigested or incompletely digested nutrients entering the general circulation and causing an immune response due to them being recognized as foreign bodies [148,149,150]. Food that has generally been fully tolerated becomes a foreign body and may contribute to the formation of antigen–antibody complexes [151,152]. The resulting immune complexes activate the complement system. There is an increased production of pro-inflammatory factors (IL-1, IL-6, TNF-α), free radicals and proteases. The emerging inflammatory process stimulates the secretion of subsequent doses of cortisol. The abovementioned phenomenon is seen as a trap—the alternating inflammation coexists with the progressive disintegration of the intestinal barrier [26,153]. Accordingly, an additional element involved in communication between the gut and the brain is the immune system cells that produce inflammatory cytokines. Immune cells are mainly found in the vermiform and in the wall of the small intestine and large intestine, however, the lymph nodes in the mucosa of the small intestine harbor the greatest amount of them. Dendritic cells, which are cells of the lymphatic system, are in contact with projections of microorganisms living near the intestinal wall [147]. When determining the functions of the microbiota and its influence on the proper functioning of the intestinal barrier in correlation with the nervous system, it is impossible to ignore the fundamental role of the vagus nerve (X nerve); responsible for transmitting signals from the brain to the intestines and from the intestines to the brain. Notably, the transmission of impulses from the intestines to the brain accounts for 90% of all body signals. These signals mainly concern the adjustment of intestinal peristalsis, its blood supply, and bile production in response to the constantly changing environment of the digestive tract. It turns out that the abovementioned importance in the transmission of signals is also related to the behavior of humans and animals. A study by S. Collins and J. Cryan showed that manipulation of the intestinal microbiota changed the behavior of animals that were subjected to tests assessing anxiety, depression and memory processes [154,155]. The mediator of the changes turned out to be the vagus nerve, the cutting of which resulted in the disappearance of the influence of microbiota on the behavior of animals.

## 6. The Influence of Diet on the Development of Depression

When assessing the multidirectionality of the factors affecting the appearance of depression, it is impossible not to discuss the significant role of diet. Studies on healthy adults show that depression occurs less frequently in people who have healthy eating patterns with a diet including a large number of fruits and vegetables, alongside a moderate intake of dairy products, eggs, fish and unsaturated fats [156,157,158]. On the other hand, separate studies conducted over ten years have shown a significant link between bad dietary habits and an increased predisposition to depression. Furthermore, such studies showed that there is also an opposite link. The perpetuation of abnormal dietary patterns is often promoted by depression [159]. The supply of certain nutrients is very important—especially in medical conditions—but it has been determined that some foods may have a preventive effect. Diet is an important variable in the relationship between diseases of the intestine and the nervous system. Dietary models showing positive effects on mental health focus on maintaining the growth of beneficial microflora, limiting the growth of pathogenic microflora and affecting intestinal barrier permeability and inflammation [97].

A great deal of research on neuropsychiatric disorders in subjects assumes the validity of the correlation between the consumed diet and the occurrence of neuropsychiatric disorders. Over the years it has been noticed that patients with IBS are more strongly associated with anxiety behaviors [160]. IBS is associated with gastrointestinal disturbances and is characterized by abdominal pain and bowel dysfunction [161]. Many patients also suffer from ongoing inflammation of the intestinal mucosa which is associated with the activity of T lymphocytes and inflammatory cytokines [162]. Despite the fact that IBS is largely described by dysfunctions in the digestive system, it has been found that this syndrome affects the functioning of the gut–brain axis because psychiatric disorders are very often also found in IBS patients. IBS also affects the functioning of the intestinal microbiota. Studies by Palma and colleagues used an animal model after colonization with fecal microflora from an IBS patients with high and low levels of histamine in the urine (separate immune activity). Mice were assigned to specific diets of either low or high contents of FODMAP (fermentable oligosaccharides, disaccharides, monosaccharides and polyols—LF (low-food) and HF (high-food)). After three weeks, GI transit (ball test), cecum volume by CT (computed tomography) scan and intestinal permeability were assessed. The animals were euthanized and the excitability of the neurons was assessed on the basis of the patch clamp records of DRG neurons (Dorsal Root Ganglion Neurons; action potential reobase). Mice receiving the HF diet showed slower gastrointestinal GI transit and increased caecal volume (increased gas content) compared to LF mice. Fermentable carbohydrates contribute to changes in the functioning of the GI tract and the extent of these changes is determined by the gut microflora [163]. The translation of the relationship between the digestive and nervous systems was determined in clinical trials. The results of these analyses indicate a positive effect of cognitive-behavioral therapy, which contributes to a significant decrease in digestive and nervous system symptoms. A pilot study with a gastroenterologist, psychiatrist, and registered nutritionist showed a significant reduction in IBS symptoms over 12 weeks. It has been suggested, that a low-FODMAP diet reduced abdominal pain and gas production [164]. In the Ohland study, conducted in 2013, the influence of diet on the occurrence of mental disorders was confirmed [165]. The experiment used an animal model with a physiological genotype and animals lacking the IL-10 gene, which showed increased susceptibility to infections. Therefore, it was proven that a Western-style diet contributes to the increased susceptibility to anxiety behavior and adversely affects the memory profile. In addition, supplementation with *Lactobacillus helveticus* in IL-10-/-mice significantly reduced the negative impact of the above diet on the intensification of anxiety disorders. Additionally, memory performance also improved following supplementation. Other studies have also determined that a Western-style diet, characterized by the consumption of increased amounts of refined sugar, contributed to increased susceptibility to inflammatory bowel disease (IBD). This action possibly occurs through changes in the composition and/or functioning of the gut microbes. In addition, a reduction in the proportion of SCFA was also noted, including acetate and butyrate, which are produced from indigestible fibers by microbial fermentation and are essential for intestinal homeostasis [166].

A number of studies have indicated that lowering the total carbohydrate content of the diet reduces the number of *Eubacteriumrectale*. This is a butter-producing group of bacteria [167]. As mentioned in the above chapter, buttermilk plays a varied role, containing molecules of an anti-inflammatory nature and ensuring the tightness of the intestinal barrier. A separate study showed that feeding mice with plenty of sucrose significantly reduced the abundance of *Bacteroidetes*, while this increased the number of *Proteobacteria, Firmicutes* and pathogenic *Helicobacteraceae* [168]. Plant-based diets can increase SCFA, along with elevated levels of *Prevotella* [169].

Dairy plays an important role in the diet and it is believed that fermented milk contains a wealth of probiotic cultures. Strains contained in dairy products show beneficial health-promoting effects [170]. In addition, potential psychobiotic effects have been described in a number of studies focusing mainly on lactic acid and *Bifidobacteria*. Psychobiotic potential was firstly demonstrated in *Lactobacillus casei* Shirota. Benton at al. measured the effect of fermented milk on mood and cognitive function using *Lactobacillus casei* Shirota (1 × 10^8^ CFU (colony-forming unit)/mL). The researchers found an overall improvement in mood [171]. This strain was also the subject of a research by Kato-Katoaka et al. in 2016 [172]. In this study, students under academic stress were included. Students consumed a fermented milk product to deliver *L. casei* Shirota. The study reported that the intake of 100 mL of fermented milk containing *Lactobacillus casei* Shirota (>1 × 10^9^ CFU/mL) increased serotonin levels in feces over 8 weeks compared to a placebo. Furthermore, daily intake of the fermented milk significantly reduced the total number of days when physical symptoms occurred in response to stress [172]. Numerous studies using fermented products have yielded further conclusions. Tillisch et al. investigated the link between the consumption of fermented milk by healthy women and responses to tasks related to emotional attention [173]. Fermented milk contained *Bifidobacterium animalis* (1.25 × 10^10^ CFU), *Streptococcus thermophilus*, *Lactobacillus bulgaricus* (1.2 × 10^9^ CFU) and *Lactococcus lactis* subsp. *lactis* (1.2 × 10^9^ CFU). After 4 weeks of regular consumption of 125 g of the fermented milk, fMRI (functional magnetic resonance imaging) was carried out. The preparation contributed to the modulation of reactions of the vast brain network. The authors also noticed an influence on the activity of regions of the brain that control the central processing of emotions and sensations [173]. Recent studies by Butler et al. are also worthy of note. Such studies investigated the effects of unpasteurized dairy products on the microbiota [174]. Within 12 weeks, the participants consumed organic products, including unpasteurized milk and dairy products. Before and after, subjects provided fecal samples and completed self-assessment questionnaires, such as the Perceived Stress Scale (PSS), Hospital Anxiety and Depression Scale (HADS), Pittsburgh Sleep Quality Index (PSQI) and International Physical Activity Questionnaire (IPAQ). Stress and anxiety levels were found to have decreased significantly in people with higher baseline PSS and HADS-A results. Furthermore, *Lactobacilli abundance* and SCFA were also observed in participants consuming dairy products [174].

In the treatment of memory disorders and depression, the use of fermented soy products have elicited positive influences. In one of these studies, researchers administered soy-based milk fermented by *Lactobacillus brevis* FPA 3709 (1 × 10^6^ CFU/mL). The authors studied antidepressant effects in Sprague–Dawley (SD) rats. After 28 days of drinking black fermented soy milk, the rats were subjected to a forced swimming test. The results showed that fermented soy milk enriched with GABA had an antidepressant-like effect on fluoxetine, which is an antidepressant [175]. In addition, a recent study identified the neuroprotective and antioxidant potential of fermented *Laminaria japonica*. Patients receiving 1.5 g/day of fermented *Laminaria japonica* for 6 weeks achieved higher scores in a Mini-Mental State Examination (MMSE) test, numerical memory test, Raven test and iconical memory assessment compared to the control group [176].

It is worth noting, that supplementation with psychobiotics contributes to alleviating often-demonstrated behavioral disorders. At the same time, both naturally occurring foods and a diet rich in fermented products both represent sources of probiotics with the potential to affect the intestinal microbiome.

## 7. The Role of Psychobiotics in the Prevention and Treatment of Depression

Probiotic bacteria that, when consumed in appropriate amounts, have a beneficial effect on mental health are called psychobiotics. This name was first used by J.F. Cryan in 2013 [177]. Psychobiotics positively affect the parameters of the intestinal barrier and modulate the immune response in the GALT (gut associated limphoid tissue) area, which is involved in the development of inflammation. The beneficial effects of psychobiotics include reductions in cortisol levels and the activity of the HPA axis, as well as the modulation of vagal nerve stimulation [178]. In 2006, the first probiotic preparation containing *Lactobacillus helveticus* R0052 and *Lactobacillus rhamnosus* R0011 strains, in which a chronic psychological stress model was used, was tested. Zareie et al. [9] conducted the study using a test involving a lack of access to water. They showed that taking the above probiotic preparation during the period of stress reduces the adhesion of pathogenic bacteria to enterocytes and limits their translocation to the mesenteric lymph nodes. These observations were confirmed in the following year, when, based on the separation of the offspring from the mother and the study of animal behavior, it was determined that the probiotic supplementation had a positive effect on the integrity of the intestinal barrier [179]. In 2008, Professor Desor from France, at the first International Conference on Microbiota and the Gut–Brain Axis in Quebec, presented observations that during increased stress (the so-called defense burying test), rats given *Lactobacillus helveticus* R0052 *Bifidobacterium longum* R0175 strains behaved in a similar way to animals given sedative diazepam [180]. In 2010, Gareau et al., using a preparation containing *L. helveticus* R0052, proved that probiotics affect the HPA axis during the action of stressors. In the following years, through in vivo studies using the strategy of infecting mice and rats with intestinal pathogens and then examining their behavior, they determined that the composition of the intestinal microbiota affects the behavior of the tested animals [181]. In 2011, by conducting studies with germ-free animals infected with the intestinal pathogen *Citrobacter rodentium* and by using the water deprivation test, Gareau et al. determined that the administration of probiotics prevented the memory disorders that occurred in the infected animals subjected to the lack of water stressor [181]. This work was published in 2011 [182].

In 2009–2012, a team led by Professor Rousseau from the University of Montreal used a model of myocardial infarction in rodents [183]. The production of pro-inflammatory cytokines and apoptosis of brain cells located within the limbic system—responsible for emotions—represent the changes described in the abovementioned animal model. In the examined animals, it was observed that the permeability of the intestinal barrier increased and symptoms of depression manifested. In a 2009 study, Girard et al. determined that the prophylactic administration of *Lactobacillus helveticus* R0052 *Bifidobacterium longum* R0175 strains before the onset of infarction in rats reduces the severity of programmed cell death (apoptosis) in the limbic system [184]. Another study using the abovementioned bacterial strains verified the significant effect of reducing the production of pro-inflammatory cytokines and the de-encapsulation of the intestinal barrier. Behavioral tests showed that probiotic therapy reduced the intensity of depressive behavior in rodents. Researchers concluded that the introduction of probiotics was effective in reducing behavioral deficits and processing emotional memory in the model of depression caused by myocardial infarction [185]. In 2016, Callaghan et al.—using the MS test (maternal separation)—found that administration of probiotics restored normal development and emotional stability in rats experiencing stress at an early stage of development [186]. Additionally, supplementation with *Lactobacillus helveticus* R0052 and *Lactobacillus rhamnosus* R0011 was effective in reducing the generational effects of stress in infants of rats in the F1 and F2 generations. In the Ait-Belgnaoui study from 2014, supplementation with *Lactobacillus helveticus* R0052 and *Bifidobacterium longum* R0175 and the WAT test (water loss test) were used [187]. It was determined that the administration of probiotics reduces deficits related to plasticity and neurogenesis that are caused by chronic stress. The consequence of these actions was a decrease in the activity of the HPA system and the autonomic system due to the reduced concentration of cortisol and catecholamines. It was proven that probiotics improved the tightness of the intestinal barrier, but these benefits were not observed for another probiotic species—*L. salivarus*. In the latest study from 2018, Ait-Belgnaoui, again using the water lack test, determined that the administration of probiotics with the following composition: *Lactobacillus helveticus* R0052 and *Bifidobacterium longum* R0175 significantly, reduces visceral hypersensitivity caused by chronic stress [188]. These changes additionally correlated with a decrease in corticosterone, noradrenaline and adrenaline levels.

Studies identifying the positive effects of psychobiotics have been also conducted, using separate strains of probiotic bacteria. In the studies by Liu et al., naïve adult mice and mice under stress early in life were given 1 × 10^9^ CFU *Lactobacillus plantarum* PS128 [189]. An open-field test determined that the supply of *Lactobacillus plantarum* PS128 increased psychomotor activity in both stressed and naïve adult mice early in life. In the cross labyrinth test, *Lactobacillus plantarum* PS128 helped reduce anxiety behavior in the naïve adult mice. These activities were not observed in stressed mice early in life. The study identified a noticeable effect on immune system parameters. The supply of psychobiotics used in the study reduced serum corticosterone levels in both primary and stressful states in mice that were stressed early in life. However, no effects on naïve mice were noticed. Levels of inflammatory cytokines were also decreased and anti-inflammatory cytokines in the serum of mice stressed early in life increased. Levels of neurotransmitters in the CNS were also studied. Dopamine levels in the prefrontal cortex were elevated in both treatment groups, while serotonin levels were only elevated in the naïve adult mice [189].

Another new study confirming the beneficial effects of psychobiotics in animal models of depression was that conducted by Tian et al. [190]. The researchers identified the psychobiotic potential of *Bifidobacterium breve* CCFM1025. In the experiment, C57BL/6J mice were given 0.1 mL/10 g body weight suspension *Bifidobacterium breve* CCFM1025 at a concentration of 1 × 10^9^ CFU/mL per day for 5 weeks. The study found that treatment with *Bifidobacterium breve* CCFM1025 helped to reduce anxiety and depressive behaviors. Inflammation caused by HPA axis reactivity was alleviated. This resulted in a decrease in glucose and hypocamic receptor expression. Reduced levels of IL-6 and circulating TNF-α were also determined. In addition, an increase in BDNF regulation was noted. Supplementation with the aforementioned psychobiotic restored balance in the intestinal microflora, especially in terms of the ratio of *Actinobacteria* to *Proteobacteria* [190]. In a similar preclinical study, Hao et al. studied the potential of *Faecalibacterium prausnitzii* ATCC 27,766 [191]. A total of 60 male rats were divided into three groups: untreated rats, stress-exposed rats and stress-exposed + *F. prausnitzii* group rats. Rats were fed 200 uJ pbs salt solution, a phosphate buffered suspension containing 1 × 10^9^ CFU *Faecalibacterium prausnitzii* ATCC 27,766 daily for 4 weeks, through an oral tube. The researchers concluded that 766 supplementation alleviated symptoms of anxiety and depression. Prophylactic and therapeutic effects on symptoms of depression and anxiety in rats were indicated. The group treated with psychobiotics had higher levels of SCFA in the blind gut and higher levels of IL-10 in the plasma. *Faecalibacterium* supplementation had a preventive effect and prevented stress-related effects such as the release of corticosterone, C-reactive protein and IL-6 [191]. Another study on the same animal model used young adult rats separated from their mother. Every day., the authors administered either 1 × 10^10^ live bacterial cells *Bifidobacterium infantis* 35,624 in 100 mL of drinking water orally or citalopram [192]. The animals were then subjected to a forced swimming test and cytokine concentrations, brain monoamine levels and measurements of the central and peripheral HPA axis were determined. Normalization of the immune response, reversal of behavioral deficits and restoration of basic norepinephrine concentrations in the brain were determined [192].

New strains of bacteria have been studied in order to confirm the positive properties of psychobiotics. Bravo et al. assessed whether *Lactobacillus rhmanosus* JB-1 mediated direct effects on the GABAergic system and investigated all behaviors related to GABAergic neurotransmission and stress responses [193]. In the study, mice were administered oral *Lactobacillus rhamnosus* JB-1, containing 1 × 10^9^ CFU for 28 consecutive days. The study found that the administration of probiotics reduced corticosterone content and reduced behaviors associated with depression and anxiety [193].

In a similar study, Kantak et al. evaluated the psychobiotic effect of *Lactobacillus rhamnosus* GG [194]. Male BALB/cJ mice were given the formulation at a density of 1 × 10^9^ CFU/day for 2 weeks before being injected with the serotonin antagonist RU 24969. It is known that RU induces behaviors similar to obsessive compulsive disorder (OCD) in this particular strain of mice. The *Lactobacillus rhamnosus* GG treatment group was compared with the placebo group and the fluoxetine group. The study found that *Lactobacillus rhamnosus* GG has a normalizing effect on hyperlocomotic, stereotypical twisting, tigmotaxy and persecretive marble burying. Supplementation yielded therapeutically comparable results with fluoxetine. This medicine is the standard treatment for obsessive compulsive disorder in humans. Although the study authors did not measure biological markers, it is claimed that the effect obtained by psychobiotics was mainly due to changes in cerebral serotonin signaling similar to those observed with fluoxetine [194].

Over the years, we have seen considerable interest in and use of new probiotic strains. In 2019, Tian et al. studied the effects of probiotic treatment on depression in a mouse model [195]. Adult male C57BL/6J mice were exposed to chronic, unpredictable, mild stress for 5 weeks while taking placebo, fluoxetine as a positive control or probiotic preparation containing *Bifidobacterium longum subsp. infantis* E41 and *Bifidobacterium breve* M2CF22M7 at a concentration of 1 × 10^9^ CFU suspended in a sterile saline solution. The study found that supplementation with a probiotic preparation significantly reduced the depressive behavior of mice in the forced swimming test, sucrose preference test and lowering test levels, by increasing 5-hydroxytryptamine levels and BDNF levels in the brain. This is associated with HPA activity and a reduction in serum corticosterone levels was observed in the study [195].

Preclinical tests were carried out using multi-graft preparations. Savignac et al. studied the psychobiotic potential of *Bifidobacterium breve* 1205 and *Bifidobacterium longum* 1714 to change the behavior of BALB/c mice [196]. *Bifidobacterium breve* 1205 improved stress-related behavior in mice with congenital anxiety. The study achieved an anxiolytic effect in the elevated s cross maze and reduced weight gain, suggesting a role in overall anxiety and metabolism. The mass of the spleen was altered by both escitalopram and *Bifidobacterium breve* 1205, while other parameters remained the same. *Bifidobacterium longum* 1714 reduced stress-induced hyperthermia and antidepressant-like behavior in the tail suspension test, suggesting a positive role in sensitivity to acute stress and depression [196].

This preclinical study, with promising results, quickly yielded research in humans. Allen et al. determined whether regular consumption of *Bifidobacterium longum* 1714 contributes to the effects on stress responses, cognitive function and brain activity in volunteers [197]. To exclude the effects of individual differences between variables, a pattern of repeated measurements was used. Each participant took a placebo for four weeks followed by a probiotic supplementation for another four weeks at a dose of 1 × 10^9^ CFU. Volunteers performed cognitive tests, underwent resting encephalopathy, and performed a cold pressure test at the beginning of the study, after placebo and after psychobiotics. Psychobotic supplementation was found to have undermined the increase in cortisol production and subjective anxiety in response to a socially assessed test. The study also noted a subtle improvement in hippocampus-dependent visual–spatial memory performance and increased electroencephalographic mobility of the anterior midline after psychobiotic ingestion [197].

Successful preclinical outcomes have also translated into clinical trials. In a more complex experiment, Takada et al. investigated the effects of the *Lactobacillus casei* Shirota strain on enterocerebral interactions under stress in both human and animal models [198]. For animal studies, adult male F344 rats received 3 x 10 ^9^ CFUs per feed per day or placebo for a period of 2 weeks. The rats were then subjected to a stress test to avoid water. *Lactobacillus casei* supplementation was observed to significantly suppress an increase in corticosterone levels after exposure to a stress test for avoiding water. In the clinical part of the study, healthy medical students preparing for an important exam were given *Lactobacillus casei* Shirota fermented milk (1 × 10^9^ CFU/mL) or an unfermented milk placebo for 8 weeks in a double-blind, placebo-controlled study in parallel groups. During the experiments, the participants were asked to complete daily and weekly questionnaires on any physical discomfort. Additionally, saliva samples were taken at the beginning of the study, 6 weeks after the intervention, on the eve of the study, and immediately after on the same day as the test to measure cortisol levels. The authors observed that the change in cortisol from the baseline was significantly lower in the psychobiotic group than in the placebo group on the day before the study. As regards the incidence of physical symptoms, the *Lactobacillus casei* Shirota supplemented group had a significantly lower incidence of influenza symptoms and abdominal symptoms compared to the placebo group, which once again showed a positive role for psychobiotics [198].

Promising results in animal model studies cannot be overlooked, thus, clinical trials soon followed. In 2008, it was shown that a probiotic preparation containing *Lactobacillus helveticus* R0052 and *Bifidobacterium longum* R0175 strains reduced stress-induced digestive ailments in healthy people. This study was placebo-controlled, randomized, and double-blinded. Probiotics were administered for 3 weeks [199]. Another study was conducted in humans in 2010 with a very similar design: placebo-controlled, randomized, and double-blinded. It used validated psychological scales to assess emotional dysfunctions, such as: anxiety, depression, the ability to cope with a stressful situation, and the measurement of cortisol levels. Cortisol levels can be assessed in the urine or in the blood by measuring the concentration of free cortisol in a 24 h urine collection or in the blood or saliva at specified times. The probiotic was administered for a period of four weeks. There was a reduction in cortisol levels compared to in those who received the placebo. Additional aspects identified in the study include: a reduction in the intensity of anxiety and depressive disorders, as well as a general improvement in the ability to cope with stressful situations. Studies carried out after one year in this group of patients showed a persistent low concentration of cortisol. On the basis of the obtained results, the authors of the study concluded that probiotics are effective in cases where people are experiencing mild stress. According to researchers, supplementation with probiotics represents a preventive measure aimed at ensuring the proper functioning of the digestive tract and reducing unpleasant psychological symptoms related to stress. [180,199]. In a 2017 study, 79 patients with at least moderate mood disorders who had declared that they had not taken psychotropic drugs, were randomly assigned to a probiotic or placebo group. After two months of study, no significant differences were found between the groups in terms of the assessment of emotional state or the concentrations of the biomarkers studied (including: IL-1β, IL-6, TNF-α, vitamin D, BDNF). According to the authors, the above results may be the outcome of the intensification of emotional disorders, their chronic nature or the patient’s resistance to monotherapy with probiotics, hence, they should be treated as preliminary [200].

Psychobiotic preparations, in addition to their activity in the GI tract, can also affect the modification of the synthesis pathways of neurohormones and neurotransmitters. Steenbergen et al. tested a multispecies probiotic preparation containing *Bifidobacterium bifidum* W23, *Bifidobacterium lactis*, W52, *Lactobacillus acidophilus*, W37, *Lactobacillus brevis* W63, *Lactobacillus casei*, W56, *Lactobacillus salivarius* W24 and *Lactococcus lactis* (W19 and W58) [201]. The aim of the study was to determine whether the above strains could reduce cognitive reactivity in people who did not have depression. The researchers conducted a triple-blind, placebo-controlled, randomized evaluation project before and after the intervention. In this study, 20 healthy participants received a 4 week probiotic intervention in the form of a dietary supplement with a probiotic at a concentration of 2.5 × 10^9^ CFU/g, while 20 other participants received a placebo. In the pre-intervention and post-intervention assessments, cognitive reactivity to sad mood was assessed using the Leiden index. This is a depression sensitivity scale. Unlike the participants who received the placebo intervention, the participants who received 4 week multispecies probiotics significantly reduced overall cognitive reactivity to a sad mood. Although the researchers did not measure any biological markers to explain the possible mechanism of action behind the psychobiotic effect, they found that the intestinal microflora increases the level of tryptophan in plasma, facilitating the circulation of serotonin in the brain, which is associated with cognitive reactivity to a sad mood [202].

Positive benefits of using psychobiotics were identified in a similar study. Kazemi et al. compared the effects of probiotic and prebiotics on lowering beck depression inventory (BDI) rates in adult patients with mild to moderate MDD, through a double-blind randomized trial [201]. Researchers measured the kynurenine/TRP ratio and TRP/branched amino acids. The aim of the study was to investigate any changes in neurotransmial metabolism. In the study, 110 depressed patients were randomized to receive a 10 billion CFU probiotic, containing *Lactobacillus helveticus* R0052 and *Bifidobacterium longum* R0175, galactooligosaccharide or a placebo for 8 weeks. Serum TRP and branched chain amino acids (BCAAs) were measured using high-performance liquid chromatography (HPLC), while metabolites of the kynurenine pathway were evaluated using an ELISA (enzyme-linked immunosorbent assay) set. Supplementation with a probiotic preparation resulted in a significant decrease in the BDI score compared to placebo and prebiotics groups. There were no significant differences between the groups in terms of the serum kynurenine/tryptophan ratio and the TRP/BCAA ratio. Importantly, the ratio of kynurenine/TRP decreased significantly in the probiotics group after adjustment to serum isoleucin. The ratio of TRP/isoleucine increased significantly in the probiotics group compared to the placebo group. Researchers have determined that probiotics reduce the activity of enzymes that alter TRP into kynurenine, leading to an increase in serotonin levels. Therefore, a decrease in the ratio of kynurenine/TRP may be a mechanism of the observed effect related to depression [201].

Interest in probiotics has yielded further conclusions in favor of their application for reducing anxiety disorders. In another study, to determine the effect of daily *Lactobacillus casei* Shirota supplementation on stress and anxiety in athletes, 20 male players participated in a study, in which they were given a probiotic stick containing 1 × 10^9^ CFUs of the *Lactobacillus casei* Shirota strain for 8 weeks every day. Anxiety and perceived stress were measured at the beginning of the study, at week 4, and week 8, using the anxiety inventory as a competitive state and the scale of the level of stress experienced. Daily psychobiotic supplementation was found to significantly reduce the results of anxiety as a cognitive state, somatic state, and perceived stress levels [203]. The study did not measure biological parameters, so the mechanism of action by which the strain selected in the study eliminated anxiety behavior was not identified [204].

Although psychobiotics have been shown to demonstrate positive effects, a separate study identified their effects in combination with antidepressants. Miyaokai et al. conducted an experiment to assess the effects of *Clostridium butyricum* MIYAIRI 588 as an adjunct therapy for the treatment of severe refractory depression [205]. Patients who were enrolled in the study were randomly assigned to a follow-up treatment with psychobiotic supplementation (60 mg per day) or a control group. To assess any significant changes, a 17-degree Hamilton Depression Scale, Beck’s Depression Scale (BDI), and Beck’s Anxiety Inventory (BAI) scale were used. Supplementation with psychobiotics in combination with antidepressants was shown to provide significant improvements in depression symptoms. A total of 70% of patients responded positively to treatment, with a remission rate of 35%. There was a greater than 50% reduction in the Hamilton’s 17-point depression scale, BDI scores and BAI results at the end of the 8 week study, regardless of the type of antidepressant used. *Clostridium butyricum* MIYAIRI 588 was found to be more effective even in the patients with more severe depressive disorders who failed to respond adequately to previous antidepressant therapy. Although this research did not focus on measuring changes in biological agents to explain any possible mechanism that could explain their findings, the authors conclude that *Clostridium butyricum* has a strong neuroprotective and anti-inflammatory effect [205].

In another study, Nishida et al. studied the psychobiotic potential of *Lactobacillus gasseri* CP2305 in alleviating symptoms associated with chronic stress in medical students. It should be noted, however, that the bacterial strain was inactivated before consumption by the experimental subject, which means that it was no longer considered a probiotic, but rather a parabiotic. Each student drank a daily placebo or drink containing 1 × 10^10^ bacterial cells for 12 weeks. All subjects were asked to track their physical and mental health using questionnaires such as the 28-point General Health Questionnaire, Hospital Anxiety and Depression Scale, The State Inventory and Features of Spielberger Anxiety, and the Pittsburgh Sleep Quality Index (PSQI). In addition, the students’ responses to biological stress were measured, including measurements such as basic saliva cortisol levels, electroencephalogram assessment during sleep, autonomic nervous activity test and levels of stress-responsive miRNA in circulating leukocytes. The use of psychobiotics significantly reduced the escalation of cortisol levels in saliva compared to the placebo group [206].

The latest meta-analysis, carried out in 2018 (analysis of 10 studies with 1349 participants) showed that probiotic supplementation does not reduce the severity of depression symptoms, when all participants in the 10 studies are included in the analysis. When divided into two separate groups consisting of depressed patients and healthy people, the analysis indicated that the severity of symptoms decreased in sick people (anxiety disorders, moderate/mild depression). The methodological heterogeneity that appeared in the above analysis concerned various factors, including: different duration of probiotic use, duration of supplementation, and the quantitative or qualitative composition of the preparation. The distinctness and lack of uniformity as regards the used methods for conducting the study may have resulted in the limited credibility of both patients and physicians in terms of assessing the role of probiotics in the treatment of depression [207].

## 8. Conclusions

As increases in cytokine concentrations are consequences of the action of cortisol—the stress hormone—it is believed that the inflammatory theory is very important in the pathogenesis of depression. Research on the gut microbiota is developing very quickly and it has indicated that there is a significant correlation between the functioning of the digestive, nervous and immune system. It can, therefore, be concluded, that the intestinal microbiota combines all theories related to the pathogenesis of depression. The above justification is realized through the involvement of microbiota in the synthesis of serotonin, BDNF and its significant participation in the process of ensuring the continuity of the intestinal barrier, in addition to its influence on the metabolism of tryptophan. There is also evidence that the metabolites of the gut microbiota are potentially associated with depression.

The tightness of the intestinal barrier can be disturbed by many factors. This barrier shows sensitivity to the action of immune system mediators: food. Additionally, its proper functioning depends on tryptophan concentrations. The abovementioned factors may individually or jointly adversely affect the tightness of the barrier. When translating the above factors into the area of depression, a certain similarity is noted as the disease is associated with changes in the immune and digestive systems, as well as the metabolism of tryptophan. The correct functioning of the gut–brain axis is, therefore, associated with a multidirectional relationship. The assumption that determines the effect of intestinal membrane disintegration on an increased predisposition to depression can also be viewed the other way around, as depression may contribute to changes in the intestine by prior activation of the immune system and its impact on the structure of the intestinal epithelium. Currently, the effectiveness of the treatment of depression is ensured primarily by psychotherapy and/or rational pharmacotherapy prescribed by a doctor, with the necessity to adapt specific recommendations to the patient. Introducing probiotics as adjuvants to treatment could improve the function of the gastrointestinal tract and mood; effects which have been observed in many studies. However, it should be noted that despite the evidence proving their effectiveness in both preventing and treating depression, these products do not currently have the status of antidepressants. Both naturally occurring foods and a diet rich in fermented products constitute sources of probiotics that can affect the intestinal microbiome. The authors express the hope that the present work can be used to consider the development of new therapeutic strategies for this disease, taking into account the described dependencies in the pathomechanism of its formation.

## Data Availability

Acquainted.
